# Highly Sensitive and Flexible Capacitive Pressure Sensors Combined with Porous Structure and Hole Array Using Sacrificial Templates and Laser Ablation

**DOI:** 10.3390/polym16162369

**Published:** 2024-08-21

**Authors:** Yibin Zhao, Jingyu Zhou, Chenkai Jiang, Tianlong Xu, Kaixin Li, Dawei Zhang, Bin Sheng

**Affiliations:** 1 School of Optical Electrical and Computer Engineering, University of Shanghai for Science and Technology, Shanghai 200093, China; 2135050829@st.usst.edu.cn (Y.Z.); 213330579@st.usst.edu.cn (J.Z.); 15003438539@163.com (C.J.); 223330649@st.usst.edu.cn (T.X.); 233350670@st.usst.edu.cn (K.L.); dwzhang@usst.edu.cn (D.Z.); 2 Shanghai Key Laboratory of Modern Optical Systems, Engineering Research Center of Optical Instruments and Systems, Shanghai 200093, China

**Keywords:** flexible capacitive pressure sensor, porous structure, array of holes, polymer, laser ablation

## Abstract

Flexible, wearable pressure sensors offer numerous benefits, including superior sensing capabilities, a lightweight and compact design, and exceptional conformal properties, making them highly sought after in various applications including medical monitoring, human–computer interactions, and electronic skins. Because of their excellent characteristics, such as simple fabrication, low power consumption, and short response time, capacitive pressure sensors have received widespread attention. As a flexible polymer material, polydimethylsiloxane (PDMS) is widely used in the preparation of dielectric layers for capacitive pressure sensors. The Young’s modulus of the flexible polymer can be effectively decreased through the synergistic application of sacrificial template and laser ablation techniques, thereby improving the functionality of capacitive pressure sensors. In this study, a novel sensor was introduced. Its dielectric layer was developed through a series of processes, including the use of a sacrificial template method using NaCl microparticles and subsequent CO_2_ laser ablation. This porous PDMS dielectric layer, featuring an array of holes, was then sandwiched between two flexible electrodes to create a capacitive pressure sensor. The sensor demonstrates a sensitivity of 0.694 kPa^−1^ within the pressure range of 0–1 kPa and can effectively detect pressures ranging from 3 Pa to 200 kPa. The sensor demonstrates stability for up to 500 cycles, with a rapid response time of 96 ms and a recovery time of 118 ms, coupled with a low hysteresis of 6.8%. Furthermore, our testing indicates that the sensor possesses limitless potential for use in detecting human physiological activities and delivering signals.

## 1. Introduction

As the complexity of wearable systems [1,2,3] continues to advance, scholars have shown a growing interest in the advancement of flexible and wearable pressure sensors [4]. The utilization of these sensors has experienced a notable increase in various applications, including medical monitoring [5,6,7,8], human–computer interactions [9,10,11,12], electronic skins [13,14,15], and other fields, owing to their exceptional sensing capabilities, compact dimensions, and robust shape retention properties, among other benefits. Wearable pressure sensors can be classified based on their operating principles as piezoresistive [16,17], capacitive [18,19,20], friction electric [21,22], and piezoelectric [23] pressure sensors. Capacitive pressure sensors are distinguished among various sensor types for their straightforward manufacturing process, minimal energy consumption, superior stability, and rapid response time [20,24,25,26]. Previous studies have indicated that conventional capacitive sensors primarily utilize solid silicone rubbers such as polydimethylsiloxane (PDMS) [27,28] and ecoflex [29]. However, despite the flexibility of these silicone rubbers, their low compressive strain and high Young’s modulus [30] result in reduced sensitivity [31], thereby failing to meet the requirements for high sensitivity sensors. To improve the sensitivity of capacitive pressure sensors within a certain pressure range, scholars have suggested the incorporation of microstructures within the dielectric layer [32]. Drawing from previous research, scholars have developed microstructures in the forms of micropyramidal [33,34,35], microcylindrical [36,37,38,39], and microconical [19,40] shapes. For example, Luo et al. present a capacitive pressure sensor with a tilted micropillar array structure in its dielectric layer prepared by a photolithographic method. This sensor exhibits high pressure sensitivity (0.42 kPa^−1^) and a very small detection limit (1 Pa) [39].

Moreover, incorporating a porous structure into the dielectric layer is a method that can enhance sensitivity [41,42,43,44]. This structural modification reduces the Young’s modulus of the dielectric layer, facilitating compression of the flexible sensor and ultimately increasing sensitivity [45]. Previous research has explored different techniques for creating polymer-based porous dielectric layers. For example, sacrificial templates [46,47,48], gas foaming [49,50,51], 3D printing [52,53], and other methods [54,55] have been used in various studies. The sacrificial template method is a popular choice due to its ease of use and high effectiveness. The preparation process entails blending removable particles with silicone rubber prior to its curing, allowing for the embedding of particles within the cured silicone rubber. These particles facilitate the formation of interconnected air exchange channels [41]. Pores can be generated in the silicone rubber by extracting the particles. Salt and sugar [47,48] are commonly used as sacrificial templates due to their high water solubility, facilitating their removal. In addition to these conventional templates, researchers are actively investigating alternative sacrificial templates such as polystyrene (PS) beads [56,57]. Yang et al. obtained porous micropyramidal structured dielectric layers by pressing PDMS into a micropyramidal silicon mold filled with PS beads and curing it, followed by dissolving away the PS beads using toluene. Capacitive pressure sensors which were based on this dielectric layer exhibit an extremely high sensitivity of up to 44.5 kPa^−1^ in the pressure range of 0–100 Pa [33].

The susceptibility of the dielectric layer to compression at low pressures, resulting in rapid saturation of the sensor and a reduced operating range, hinders its ability to accurately detect higher pressures. Additionally, repeated compressions may lead to destruction of the microstructures due to interaction forces between them. In order to combine high sensitivity with a wide pressure detection range and to maintain good recovery properties of the dielectric layer, Li et al. obtained a highly porous dielectric layer by the sacrificial NaCl template method, while the insertion of multiple metal pins introduced a through-hole array in the porous dielectric layer, thus further improving the porosity. This working design of the sensor achieves a sensitivity of 1.15 kPa^−1^ within 0–1 kPa and the device has a very wide operating ranging from 5 Pa to 1 MPa [45]. However, damage to the dielectric layer during metal pin demolding can compromise sensor performance. Jiang et al. demonstrate the fabrication of microstructures with uniform geometry and adjustable size through laser ablation on a dielectric substrate [40]. This cost-effective and efficient method offers a practical approach for designing arrays.

This research presents the fabrication of an innovative flexible capacitive sensor that boasts a distinctive design. Notably, this novel sensor incorporates a dielectric layer characterized by a porous architecture and an array of holes, setting it apart from conventional sensors. The porous dielectric layer was fabricated using the sacrificial NaCl template method, while the hole array was created through laser ablation of the porous dielectric layer. By sandwiching this porous dielectric layer with the hole array between flexible electrodes made of polyimide tape and copper foil, a flexible capacitive pressure sensor was developed. The sensor exhibited a notable sensitivity of 0.694 kPa^−1^ within the 0–1 kPa range, while demonstrating a broad pressure response spanning from 0 to 200 kPa. Experimental evaluations revealed its capability to discern pressures as minute as 3 Pa, accompanied by remarkable consistency across 500 high-pressure cycles, underscoring its reliability and durability. Furthermore, the sensor’s utility is showcased in monitoring human physiological activities, such as swallowing and elbow flexion, as well as in Morse code communication.

## 2. Materials and Methods

### 2.1. Materials

Polydimethylsiloxane (PDMS) and a silicone elastomer curing agent were purchased from Dow Corning in Midland, MI, USA. Sodium chloride (NaCl microparticles, ≥99.5%, with a particle size distribution in the range of 5–70 µm) was purchased from China Salt Shanghai Salt Industry Co., Ltd., in Shanghai, China. The flexible electrodes were manufactured using copper foil purchased from Anhui Zhengying Company in Fuyang, Anhui, China, and polyimide (PI) tape from Hangzhou Ubisoft Company in Hangzhou, Zhejiang, China.

### 2.2. Preparation of Porous PDMS Dielectric

**Figure 1:**
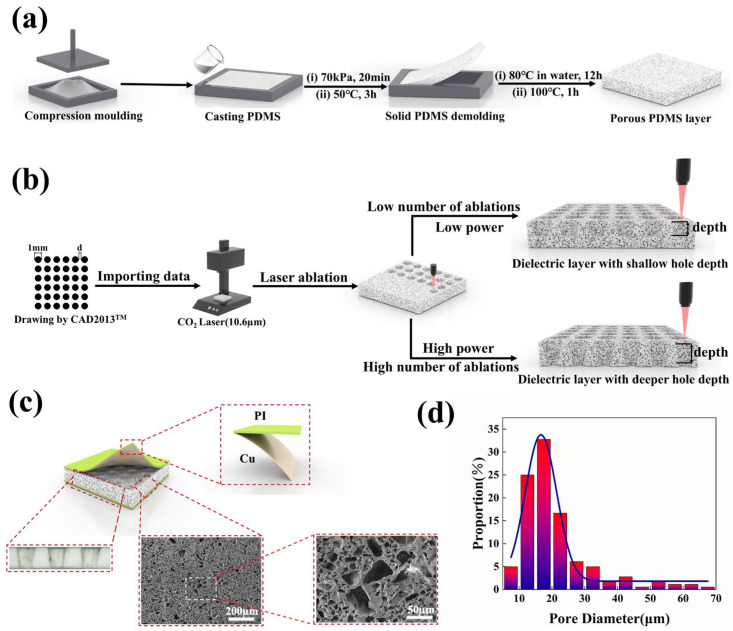
(**a**) Schematic illustration of the process for preparing a porous PDMS dielectric layer. (**b**) A detailed schematic representation of the laser ablation process. (**c**) A visual representation of the flexible capacitive pressure sensor, including an optical microscope view of the hole array profile in the bottom left panel and scanning electron microscopy images of microporous structures in the bottom two panels on the right. (**d**) Graph depicting the distribution of micropore sizes within the porous structures of the dielectric layers.

The sacrificial template method was employed to fabricate porous dielectric layers using salt microparticles with a particle size distribution ranging from 5 to 70 µm. The procedure for creating the porous PDMS dielectric layer is illustrated in Figure 1a. Specifically, 5.521 g of salt was introduced into a mold measuring 60 mm in diameter and 2 mm in height and then compressed to conform to the mold shape. Subsequently, 3.110 g of PDMS prepolymer was introduced into the templates, and the molds containing the PDMS prepolymer were subjected to a vacuum treatment in a vacuum machine (LC-DZF-6050AB, Lichen Instrument Technology Co., Shanghai, China) at a temperature of 25 °C and an air pressure of 0.07 MPa for 20 min. This process facilitated the thorough infiltration of the PDMS prepolymer into the molded salt templates. The PDMS prepolymer utilized in this study was prepared by blending PDMS with a curing agent through homogeneous stirring for a period of 20 min, with a mass ratio of PDMS to curing agent set at 10:1. After complete infiltration of the PDMS, the sample was subjected to atmospheric pressure and heated to a temperature of 50 °C for 3 h to allow the PDMS prepolymer to undergo full curing. Subsequently, the demolded sample was transferred to a water bath heating unit (DF-101S5L, Lichen Instrument Technology Co., Shanghai, China) and immersed in hot water at 80 °C for 12 h in order to eliminate salt residues. Finally, the sample was dried in a drying oven at 100 °C for 1 h, resulting in the formation of a porous PDMS dielectric layer with a porosity of approximately 46%.

### 2.3. Preparation of Porous PDMS Dielectric Layers with Hole Array

Following the preparation of the porous PDMS dielectric layer, the porous PDMS material was subjected to ablation using a carbon dioxide laser (K3020, Julong Laser Co., Ltd., Liaocheng, Shandong, China) with a wavelength of 10.6 µm, leading to the formation of the hole array. The specific procedures for this process are outlined in Figure 1b. Utilizing AutoCAD2013^TM^ software (G. 55. 0. 0), a 6 × 6 matrix of circular holes with a diameter of 1 mm was designed. The spacing (d) between two adjacent holes was identified as a key parameter for optimization purposes in our research. Subsequently, the designed arrays were fed into the laser machine, where the samples underwent ablation with precise adjustments of laser power and scanning speed, resulting in the creation of a PDMS dielectric layer containing an array of holes. The ablation depth of porous PDMS using varying laser powers at a consistent scanning speed is illustrated in Figure S1.

It is important to acknowledge that achieving greater hole depth at lower power levels necessitates multiple ablations to meet the specified criteria, resulting in a more time-intensive process. Elevated power levels raise the ambient temperature of the laser output beam, potentially causing “over-burning” of the hole arrays and impacting their morphology [58]. In this study, a laser power of 30 W and a scanning speed of 200 mm/s was utilized to process the porous PDMS samples.

Furthermore, the depth of the hole array can be controlled by adjusting the number of ablations. When utilizing a power of 30 W and a scanning speed of 200 mm/s, it was observed that two ablations of the porous PDMS film resulted in a hole depth of approximately 2 mm, which corresponds to the thickness of the sample films prepared.

### 2.4. Experimental Setup

The morphology of the dielectric layer was analyzed using optical microscopy (RY001, Ksgaopin, Kunshan, Jiangsu, China) and scanning electron microscopy (JSM-IT500HR, Japan Electronics, Tokyo, Japan). The porous PDMS dielectric layer, measuring 8 mm × 8 mm × 2 mm (2 mm thick), containing an array of holes, was positioned between two flexible electrodes, each measuring 8 mm × 8 mm × 0.025 mm. These electrodes were composed of PI tape (0.02 mm thick) and copper foil (5 µm thick). Due to the pressure sensitive adhesive (PSA) on the purchased copper sheet, it possessed some adhesive properties. The PSA on the electrodes was bonded to thin (50 µm thick) layers of PDMS precursor (with a 10:1 mass ratio of PDMS to curing agent), which had been pre-coated (using the scratch-coating method) on the upper and bottom surfaces of porous PDMS dielectric layers with the hole array. After the PDMS precursor is cured, the flexible electrodes can be firmly connected to the dielectric layer, ensuring no detachment occurs during compression and bending. The sample was mounted on a 5 cm diameter disk and secured to the testing apparatus using PI tape to maintain flatness and optimal contact during testing. A manual press (HLD, Handpi, Yueqing, Zhejiang, China) was utilized to exert pressure on the sensor, while a digital force gauge (HP-20, Handpi, Yueqing, Zhejiang, China) was employed to measure the real-time pressure value. The digital force gauge boasts a reading accuracy of 0.001 N and a range of 0–20 N. Copper wire was employed to connect the two electrodes of the sensor with the LCR bridge (TH2822D, Tonghui, Changzhou, Jiangsu, China). The real-time capacitance data of the sensor were recorded using the LCR bridge at a temperature of 25 °C. The data acquisition frequency of the bridge was set at 100 kHz. Afterwards, the collected data were compiled and analyzed on a computer system. The circuit connection diagram for the test of the capacitive flexible pressure sensor developed in this study is illustrated in Figure S3.

## 3. Results and Discussion

### 3.1. Measurement of Porosity in Porous Dielectric Layers

The mass of multiple prepared porous PDMS samples was individually recorded in order to improve the accuracy of porosity estimation. Subsequently, these samples were immersed in separate beakers filled with water for 8 h to ensure complete penetration of water into the porous PDMS samples. After taking these samples out of the water, the mass of each sample post-water absorption was measured and documented. The porosity of each sample was then calculated using Equation (1) [59].

(1) $$P=\frac{\left(m_{w}-m_{d}\right)/\rho_{w}}{\left(m_{w}-m_{d}\right)/\rho_{w}+m_{d}/\rho_{d}}\times 100\%$$

where $m_{d}$ is the mass of the initial porous dielectric layer, $m_{w}$ is the mass of the porous dielectric layer after sufficient water absorption, $\rho_{d}$ is the density of the PDMS, and $\rho_{w}$ is the density of water. The porosity of the porous PDMS dielectric layer, which was fabricated using the sacrificial NaCl template method, was determined to fall within the range of 46% ± 0.7% based on Equation (1).

### 3.2. Characterization of Flexible Capacitive Sensors

The capacitive flexible pressure sensor developed in this study comprises two flexible electrodes composed of flexible PI tape (0.02 mm thick) and copper foil (5 µm thick), as well as porous PDMS dielectric layer which incorporates an array of holes. Figure S2 illustrates the external appearance of the sensor and highlights its remarkable flexibility. Meanwhile, Figure 1c provides a comprehensive illustration of the flexible capacitive pressure sensor’s composition, complemented by optical and scanning electron microscope images that reveal the cross-section of the dielectric layer in detail. Analysis of the sensor dielectric layer’s cross-section reveals that the laser-ablated holes exhibit a slight tilt angle on their sidewalls, deviating from perfect verticality. The analysis of the cross-sectional intensity distribution of the laser output power reveals a Gaussian function pattern [58,60], indicating that the highest energy concentration is located at the center of the beam. Consequently, materials positioned near the beam’s center experience complete ablation during the ablation process, whereas those situated at the periphery of the beam exhibit lower energy levels and, consequently, reduced ablation efficiency. This discrepancy in energy distribution results in a slightly inclined sidewall formation. Scanning electron micrographs of the cross sections provide visual representation of the dimensions and spatial arrangement of micropores. It is observed that, with the exception of a few larger pores resulting from larger salt particles, the variation in pore size among the remaining pores is minimal. Analysis of the particle size distribution shown in Figure 1d reveals that the majority of pores fall within the range of 8–32 µm.

### 3.3. Sensing Mechanism of Capacitive Pressure Sensors

A capacitive pressure sensor’s capacitance is determined by the effective overlap area of the electrodes and the dielectric layer (A), the relative permittivity of the dielectric layer ($\varepsilon_{r}$), and the distance between the electrode plates (d). Specifically, it is determined according to the conventional capacitance formula, Equation (2), for a parallel flat capacitor.

(2) $$C=\frac{\varepsilon_{0}\varepsilon_{r}A}{d}$$

**Figure 2:**
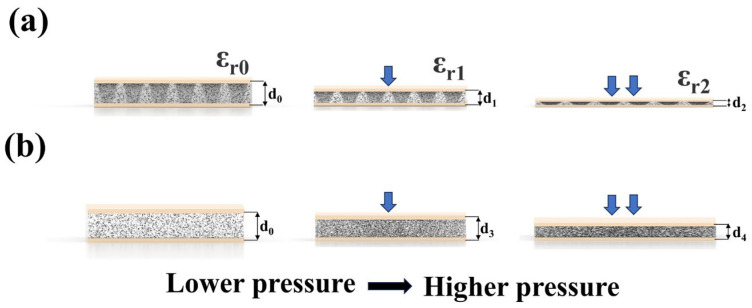
(**a**) Illustration of the workflow for a porous flexible capacitive pressure sensor, featuring an array of holes structure. (**b**) A schematic diagram elucidating the sensing mechanism of a flexible capacitive pressure sensor, featuring a porous dielectric layer without an array of holes structure.

In this equation, $\varepsilon_{0}$ represents the dielectric constant of air, while $\varepsilon_{r}$ stands for the relative dielectric constant of the dielectric layer, and d denotes the distance between the two electrode plates. In the case of a porous dielectric layer containing an array of holes, a fraction of the dielectric layer’s volume is initially filled with air. Upon application of force, the air-filled pores within the dielectric layer gradually collapse and are substituted with solid PDMS material. This process alters the sensor’s geometry, leading to variations in the dielectric constant ($\varepsilon_{r}$) and thickness (d), consequently impacting the capacitance. The sensor’s response to pressure is determined by monitoring the relative changes in capacitance. Additionally, the close and secure attachment of the flexible electrodes to the dielectric layer serves to mitigate noise interference. The electrodes employed in this study exhibit superior conductivity and flexibility, thereby enhancing the precision of data collection within narrow pressure ranges. The sensing mechanism of the sensor we designed is illustrated in Figure 2a. The dielectric layer’s relative dielectric constant is influenced by both air and PDMS due to the presence of microporous pores and the hole array. This relationship can be quantitatively determined using Equation (3).

(3) $$\varepsilon_{r}=\varepsilon_{a}v_{a}+\varepsilon_{c}v_{c}$$

The relative permittivity of air, denoted as $\varepsilon_{a}$ and approximately equal to 1, and the relative permittivity of PDMS, denoted as $\varepsilon_{c}$, are key parameters in the analysis of the dielectric layer. The volumes occupied by air ($v_{a}$) and PDMS ($v_{c}$) within the dielectric layer play a crucial role in the compression sensing mechanism of the sensor. When the dielectric layer undergoes compression, a portion of the air is displaced by PDMS, resulting in changes to both $v_{a}$ and $v_{c}$, ultimately leading to variations in the relative permittivity ($\varepsilon_{r}$).

The diagram in Figure 2a illustrates the workflow of a porous PDMS dielectric layer containing an array of holes. The initial thickness of the sensor’s dielectric layer is denoted as $d_{0}$, with a relative dielectric constant of $\varepsilon_{r0}$. The pressure response of the sensor can be categorized into two distinct phases as pressure increases from 0. During the initial phase, the sensor’s high density of micropores and the hole array results in a significant amount of air being trapped within the sensor, leading to the low Young’s modulus of the dielectric layer, which facilitates easy compression of the sensor. During the low-pressure phase, there is a rapid increase in $\Delta d_{1}=d_{1}-d_{0\;}$, as the relative permittivity of the dielectric layer transitions from $\varepsilon_{r0}$ to $\varepsilon_{r1}$ due to the partial replacement of air with solid PDMS. This enhanced sensitivity of the sensor is particularly pronounced in the low-pressure regime, contributing to its improved performance in detecting subtle pressure variations. In the subsequent stage, as the pressure increases to a higher level, the microporous pores of the PDMS layer become densified, leading to a less significant change in $\Delta d_{2}=d_{2}-d_{1\;}$. The alteration in the relative dielectric constant $\varepsilon_{r}$, transitioning from $\varepsilon_{r1}$ to $\varepsilon_{r2}$, significantly influences the variation in capacitance value. This shift in relative permittivity primarily correlates with the saturation of microporous pores and laser-ablated holes. In comparison to the porous structure-only sensor depicted in Figure 2b, our analysis revealed that the porous dielectric layer with an array of holes illustrated in Figure 2a exhibited greater compression than the porous structure-only dielectric layer shown in Figure 2b when subjected to identical pressure levels; that is, $d_{0}-d_{1\;}>d_{0}-d_{3\;}$ and $d_{1}-d_{2}>d_{3}-d_{4\;}$. The notable increase in sensor sensitivity, particularly evident in low-pressure conditions, stems directly from the incorporation of a hole matrix within the porous dielectric layer. This design feature results in a reduced Young’s modulus, enabling greater compressibility and subsequently enhancing the sensor’s ability to detect even minute pressure fluctuations. The uniformly distributed micropores within the dielectric layer, in conjunction with the array of holes extending to the base, function synergistically to enhance pressure sensing across the entire operational range. During the initial phase, when the pressure was applied, the majority of the microporous pores were filled by solid PDMS, whereas only a minor proportion of the air introduced through the array of holes was filled with solid PDMS. At this stage, the microporous pores assume a primary function, while the array of holes assumes a secondary role. In the second stage, when the pressure gradually increases, the air introduced through the array of holes assumes a primary role because it still has a large volume fraction, whereas the residual microporous pores contribute a secondary function because most of the micropores were densified. The synergistic interaction between the microporous pores and the hole array is crucial in ensuring a broad operational range and high sensitivity at low pressures for the sensors. As the pressure on the sensor is gradually released, the densified pores within the dielectric layer are re-established, resulting in the restoration of the hole array to its initial height and the morphology of the dielectric layer. This observation serves as evidence of the sensor’s favorable recoverability.

### 3.4. Sensor Performance Optimization and Improvement

The specific test steps for the performance of capacitive pressure sensors are described in Section 2.4. Pressure is applied using the HLD pressure testing machine, pressure data are recorded with the HP-200 dynamometer, and capacitance data are collected using the TH2830 LCR meter. The sensitivity equation for the sensor is presented as Equation (2).

(4) $$S=\frac{\delta \left(\frac{\Delta C}{C_{0}}\right)}{\delta p}$$

where ΔC is the capacitance value C of the sensor after it has been compressed minus the initial capacitance value $C_{0}$, and p is the pressure loaded on the sensor. It is recognized that the larger the value of $\Delta C/C_{0}$ per unit pressure, the higher the sensitivity of the sensor. Based on this theory, five different sample designs were created for comparative analysis. Specifically, the samples can be categorized into five types: bulk PDMS (bPDMS), bulk PDMS with an array of holes (bPDMS-h_2_) with a hole depth of 2 mm, porous PDMS (pPDMS), porous PDMS with an array of holes depth of 1 mm (pPDMS-h_1_), and porous PDMS with an array of holes depth of 2 mm (pPDMS-h_2_). Each sample measures 8 mm × 8 mm × 2 mm (2 mm thick) and features a 6 × 6 round hole array. The manufacturing procedure entailed the repetitive ablation of bulk PDMS material to a depth of 2 mm, accomplished through five sequential cycles using a laser power setting of 30 W and a scanning velocity of 200 mm/s. To produce porous PDMS with a uniform array of 1 mm deep holes, a singular ablation step was employed, utilizing a laser intensity of 30 W and a scanning velocity of 200 mm/s. Furthermore, to create porous PDMS featuring 2 mm deep holes, a two-step ablation process was conducted under the same laser conditions, precisely reaching the bottom of the dielectric layer during the second ablation iteration. Figure S4 illustrates the variation in hole depths resulting from ablating the porous PDMS dielectric layer once (Figure S4a), twice (Figure S4b), and ablating the bulk PDMS dielectric layer three (Figure S4d) and five times (Figure S4c) using a CO_2_ laser, as per the specified laser parameters. The data indicate that a greater number of ablations are required for the bulk PDMS in comparison to the porous PDMS to achieve equivalent hole depths. This discrepancy can be attributed to the poor light absorption of the transparent bulk PDMS [61], necessitating an increased number of ablations for effective ablation.

**Figure 3:**
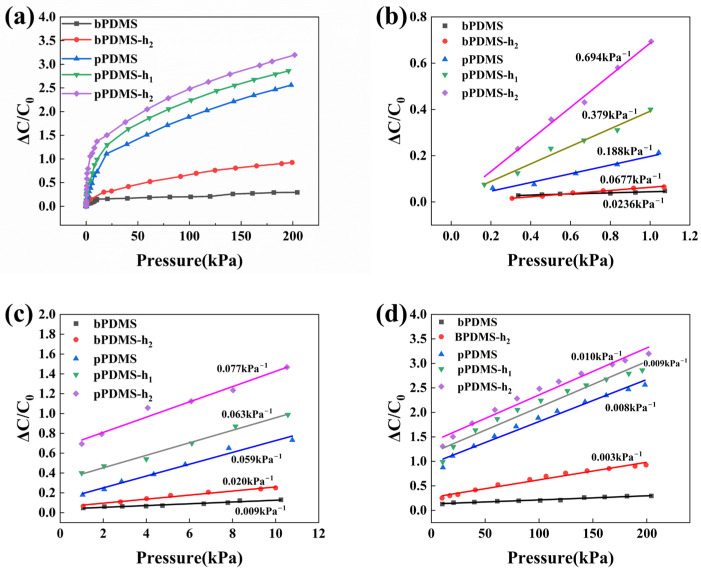
(**a**) The variation curves depicting the relationship between relative capacitance and pressure within the range of 0 to 200 kPa for five test samples. (**b**) A linear fit curve illustrating the correlation between relative capacitance change values and pressure values within the pressure range of 0 to 1 kPa. (**c**) Linear fit curves demonstrating the relationship between relative capacitance change values and pressure values within the pressure range of 1 kPa to 10 kPa. (**d**) Fit curves representing the relationship between relative capacitance change values and pressure values within the pressure range of 10 to 200 kPa.

The performance curves illustrating the relative capacitance versus pressure for the five tested sensors are presented in Figure 3. Specifically, Figure 3a displays the curves within the pressure range of 0–200 kPa. Analysis of the results indicates that the sensitivity of the sensors, namely pPDMS, pPDMS-h_1_, and pPDMS-h_2_, can be significantly improved through the incorporation of multiple micropores. This enhancement is attributed to the lower Young’s modulus of the porous samples, rendering them more compressible compared to the bulk PDMS. Despite the introduction of an array of holes in the dielectric layer of the bPDMS-h_2_ sensor, the lack of a porous structure in the dielectric layer results in all other areas being occupied by PDMS. This limits the presence of air gaps, thereby hindering the reduction of the Young’s modulus of the dielectric layer. Consequently, the dielectric layer is difficult to compress, leading to a relatively low sensitivity. Regarding the two sensors, pPDMS-h_1_ and pPDMS-h_2_, which have been modified with an array of holes in addition to the existing porous structure, their porosity is greater and their Young’s modulus is lower compared to the pPDMS sensor with only a porous structure. This results in increased compressibility and higher sensitivity of the two sensors compared to the pPDMS sensor.

To better elucidate the operational principles of the sensor, three distinct pressure intervals were selected for individual analysis of the capacitive response. In particular, the sensitivities of pPDMS-h_2_, pPDMS-h_1_, and pPDMS were determined to be 0.694 kPa^−1^, 0.379 kPa^−1^, and 0.188 kPa^−1^, respectively, within the range of 0–1 kPa, as illustrated in Figure 3b. When the applied pressure falls within the range of 1–10 kPa, the sensitivity of various sensor types diminishes, as shown in Figure 3c. However, sensors utilizing a dielectric layer with a composite structure of micropores and an array of holes still exhibit a level of sensitivity. Furthermore, the sensitivity of these sensors increases with the depth of the holes within the array. Even under extremely high pressures, pPDMS-h_2_ continues to exhibit a certain degree of sensitivity, as depicted in Figure 3d. In addition, the tests conducted yielded the relationship curves between pressure and strain in the dielectric layer for the five samples within the range of 0–200 kPa, as illustrated in Figure S5. It is evident that the sample pPDMS-h_2_, characterized by high compressibility, exhibited the highest strain of 72% at a pressure of 200 kPa, whereas bulk PDMS displayed the lowest strain of approximately 26% at 200 kPa. Additionally, all samples demonstrated strain–pressure curves that followed an exponential function increase.

The dielectric layer of a capacitive pressure sensor exhibits a capacitive response that is tied to both the material’s relative permittivity and the inter-electrode distance between its two conductive plates. Under elevated pressure, the porous structure of the dielectric layer undergoes compression, leading to the infiltration of solid PDMS into the majority of its pores. This mechanism entails a deceleration in the shrinking rate of the gap between the electrode plates, coupled with a diminished rate of growth in the relative permittivity of the dielectric layer, collectively contributing to a decrease in sensor sensitivity as the applied pressure escalates. Introducing an array of holes into the dielectric layer enhances the air content, enabling a more effortless compression response to applied pressure in contrast to a purely porous dielectric layer. This modification leads to an improved compressibility characteristic under pressure. Consequently, the sensitivity is heightened. As pressure levels escalate, the air gap within the porous dielectric layer becomes nearly solidified by the PDMS material, leading to an increase in Young’s modulus and rendering compression more challenging. Therefore, the sensitivity of dielectric layers with only microporous structures is relatively low at high pressures. Nevertheless, in the case of a porous dielectric layer containing an array of holes, the presence of a significant volume of air at the location of the hole array allows the dielectric layer to maintain an air gap even when subjected to high levels of compression. This characteristic provides the dielectric layer with the ability to respond to the higher pressures, thereby enabling sensors utilizing such porous dielectric layers to maintain sensitivity even under high-pressure conditions.

The impact of the sparsity of an array of holes in porous dielectric layers on the Young’s modulus of a sensor, and subsequently on its sensitivity, was investigated by maintaining a constant individual hole area (1 mm in diameter) and adjusting the laser ablation spacing between neighboring holes in the array. The laser ablation process results in concentrated thermal energy at the location of the holes, leading to over-burning. When the spacing between two holes is too small, the high laser energy causes an increase in material temperature outside the ablation area. This can result in the destruction of the PDMS between neighboring holes, leading to the cross-linking of the holes and compromising the intended structure of the dielectric layer. Considering the above issues and the ablation accuracy (0.06 mm) of the CO_2_ laser we used, we set the minimum pitch to 0.4 mm. The spacing was sequentially adjusted to 0.8 mm, 1.0 mm, and 1.2 mm. The depth of the holes was all 2 mm. The samples of the various classes mentioned above were named pPDMS-w_0.4_ (which is the same sample as pPDMS-h_2_), pPDMS-w_0.8_, pPDMS-w_1.0_, and pPDMS-w_1.2_. Figure S6a–c shows the physical diagrams of the pPDMS-w_0.8_, pPDMS-w_1.0_, and pPDMS-w_1.2_ dielectric layers, respectively.

**Figure 4:**
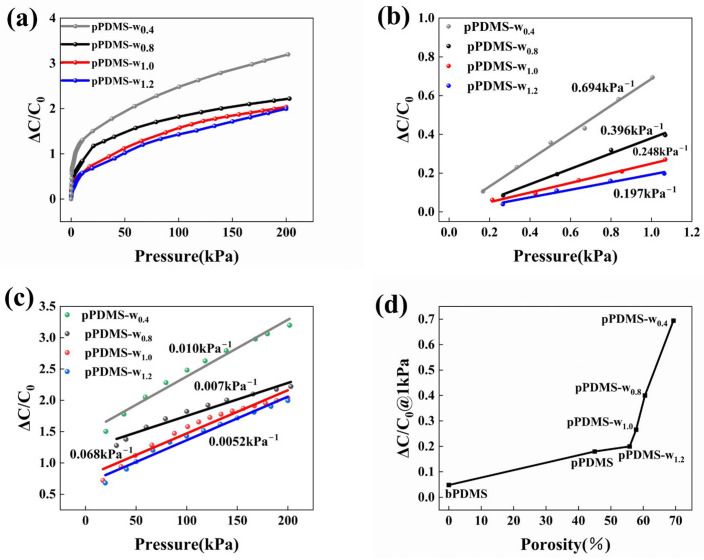
(**a**) Variation curves of relative capacitance values as a function of pressure ranging from 0 to 200 kPa for four sensors with different hole array spacings. (**b**) Linear fitting curves of relative capacitance change values versus pressure values for the four tested sensors within a pressure range of 0–1 kPa. (**c**) Linear fit curves of relative capacitance change versus pressure values for the four tested transducers for pressures ranging from 1 to 200 kPa. (**d**) Variation curve of relative capacitance with porosity at a pressure of 1 kPa.

The results of their performance are illustrated in Figure 4. Our investigation revealed that, under identical pressure conditions, sensitivity increases as the spacing of the hole array decreases. Figure 4a displays the pressure response curves of the four samples within the pressure range of 0–200 kPa. Specifically, the sensor pPDMS-w_0.4_ demonstrated the highest sensitivity of 0.694 kPa^−1^, within the pressure range of 0–1 kPa, as shown in Figure 4b. As the spacing between the hole array of various samples increases in a sequential manner, the sensitivity of the sensors correspondingly decreases across the same pressure range. The sensitivity of the sensor pPDMS-w_0.8_ is 0.396 kPa^−1^ within the pressure range of 0–1 kPa. The sensor pPDMS-w_1.0_ exhibits a sensitivity of 0.248 kPa^−1^, while the sensor pPDMS-w_1.2_ demonstrates a sensitivity of merely 0.197 kPa^−1^. It is important to highlight that despite the decrease in sensitivity of a sensor utilizing a porous dielectric layer with an array of holes, as the spacing of the array increases over the same pressure range, they remain superior to pPDMS. This is precisely the result of the increased sensitivity due to the introduction of a hole array in the porous dielectric layer, thus leading to a further reduction of the Young’s modulus of the dielectric layer. In the pressure range of 20–200 kPa (Figure 4c), the decrease in sensitivity of all four sensors is attributed to the increased density and reduced compressibility of the dielectric layer at higher pressures.

Laser ablation creates a series of perforations that introduce additional air into the porous dielectric layer, which has a porosity of approximately 46%. This process lowers the Young’s modulus of the material, facilitating compression and ultimately enhancing the sensitivity of the flexible sensor. In a specification-consistent porous dielectric layer, the volume fraction of air introduced by the hole array increases as the holes are closer together. This enhanced porosity facilitates greater compressibility of the dielectric layer, thereby improving the sensitivity of the sensor. Theoretically, the porosity of the samples can be determined by combining the air volume introduced by the sacrificial NaCl template method with the air volume introduced by the pore array created through laser ablation [45], as shown in Figure 4d. According to this theory, the porosity values for pPDMS, pPDMS-w_1.2_, pPDMS-w_1.0_, pPDMS-w_0.8_, and pPDMS-w_0.4_ are approximately 46%, 56.8%, 58.8%, 61.5%, and 69.3%, respectively. It is important to highlight that the porosity calculated through this method closely aligns with the results obtained from the porosity measurement method for porous dielectric layers discussed in Section 3.1, with a maximum discrepancy of 1%. Figure 4d illustrates the variation in relative capacitance of the sensor in relation to the porosity of the dielectric layer under a pressure of 1 kPa. The curve clearly exhibits an exponential transformation as the porosity increases. Therefore, we believe that the hole array provides an effective way to reduce the Young’s modulus of the dielectric layer and improve the responsiveness of capacitive sensors in a certain pressure range.

### 3.5. The Comprehensive Performance of Flexible Capacitive Sensors

**Figure 5:**
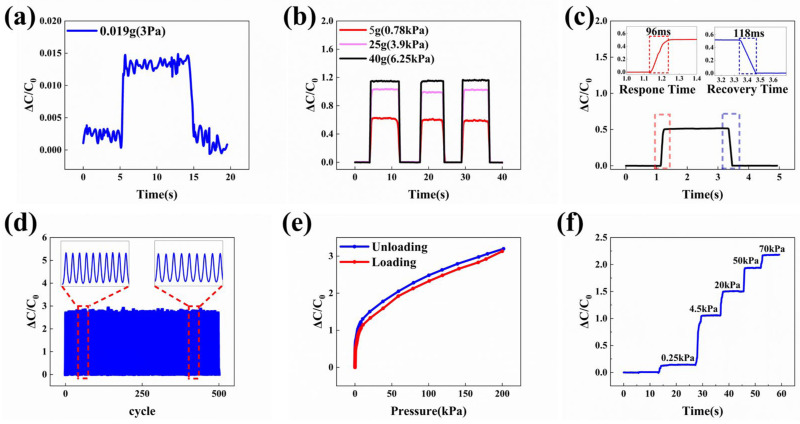
Comprehensive performance testing of capacitive pressure sensors utilizing the sample pPDMS-h_2_. (**a**) The sensor’s minimum pressure detection limit. (**b**) The sensor’s pressure response to the loading and unloading of 5 g, 25 g, and 40 g weights. (**c**) The sensor’s response time at a pressure of 0.85 kPa. (**d**) The stability test of the sensor’s responsiveness at a pressure of 105 kPa for 500 cycles. (**e**) The test of the hysteresis in the capacitive response of pressure sensors between 0 and 200 kPa during loading and unloading. (**f**) The test of the sensor’s response to stepped pressure changed between 0 and 70 kPa.

Based on the optimization process outlined above, the flexible capacitive sensor pPDMS-h_2_ was chosen for further comprehensive performance evaluations in this study. To demonstrate the sensor’s minimum detection limit, incremental pressure was applied starting from 1 Pa, resulting in a notable change in capacitance value at 3 Pa, as illustrated in Figure 5a. The sensor exhibited a significant response at 3 Pa, indicating that its minimum pressure detection threshold is approximately 3 Pa. The graphical representation in Figure 5b showcases three distinct traces, each portraying the sensor’s relative capacitance variation $\Delta C/C_{0}$ in response to static loads of 5 g, 25 g, and 40 g, respectively. It is evident that $\Delta C/C_{0}$ exhibits rapid variation during the loading and unloading, demonstrating consistent responsiveness, effective recovery, and high sensitivity. The sensor’s response and recovery times were assessed by swiftly applying and releasing a pressure of 800 Pa. Rapid application and release of pressure can be crucial for ensuring the accuracy of the measurement data. Analysis of Figure 5c reveals that the sensor achieves a response time of 96 ms when the capacitance value change rate reaches 0.58, and a recovery time of 118 ms when pressure is rapidly released. Based on this observation, the sensor’s response time aligns closely with that of human skin’s sensitivity to pressure stimuli [62], suggesting its potential applicability in monitoring human physiological activities, thereby expanding its utilization domain. To evaluate the reproducibility of the sensor’s performance, the sensor was subjected to repetitive loading and unloading cycles at a constant pressure of 150 kPa on a testing platform, totaling 500 iterations. This methodology allowed for an assessment of the sensor’s consistency over multiple cycles. The results, depicted in Figure 5d, indicate that the maximum change in samples exhibited a margin of error of ±14% throughout the cycling process. The observed maximum error value of 14% can be attributed to external interference affecting the testing machine at some point in time. Analysis of the graphical results indicates that the sensor exhibits excellent repeatability across each cycle under stable operating conditions. Consequently, it can be inferred that the sensor demonstrates enhanced durability, an extended service life, consistent responsiveness after multiple uses, and the capacity to endure high pressures. Small errors due to interference from the test equipment do not affect the overall sensing performance. The phenomenon of hysteresis, arising from the cyclic loading and unloading of the sensor within a pressure spectrum spanning 0 to 200 kPa, is visually presented in Figure 5e. The analysis reveals a maximum delay of approximately 6.8%. This lag level is nearly identical to that reported in some previous work [63,64]. This unavoidable hysteresis is due to the fact that polymers show viscoelastic behavior [65]. Figure 5f demonstrates the sensor’s exceptional dynamic pressure response within the 0–70 kPa range.

### 3.6. Performance Comparison of Different Porous Capacitive Pressure Sensors

Table 1 demonstrates some research conducted in recent years to investigate the performance of porous capacitive pressure sensors. It can be noted that the vast majority of work is developed using templates, or by dissolving away the soluble material after the elastomer layer has cured. The introduction of air gap increases the compressibility of the dielectric layer, so that the sensitivity increases when the porosity is higher. Therefore, by introducing an array of holes into the porous dielectric layer, we have obtained a higher porosity and thus improved the sensitivity of our sensors compared to other sensors with only micropores. In addition, due to the synergistic effect of micropores and a hole array, the sensor obtains a wide pressure detection range.

### 3.7. Applications Related to Flexible Capacitive Pressure Sensors

**Figure 6:**
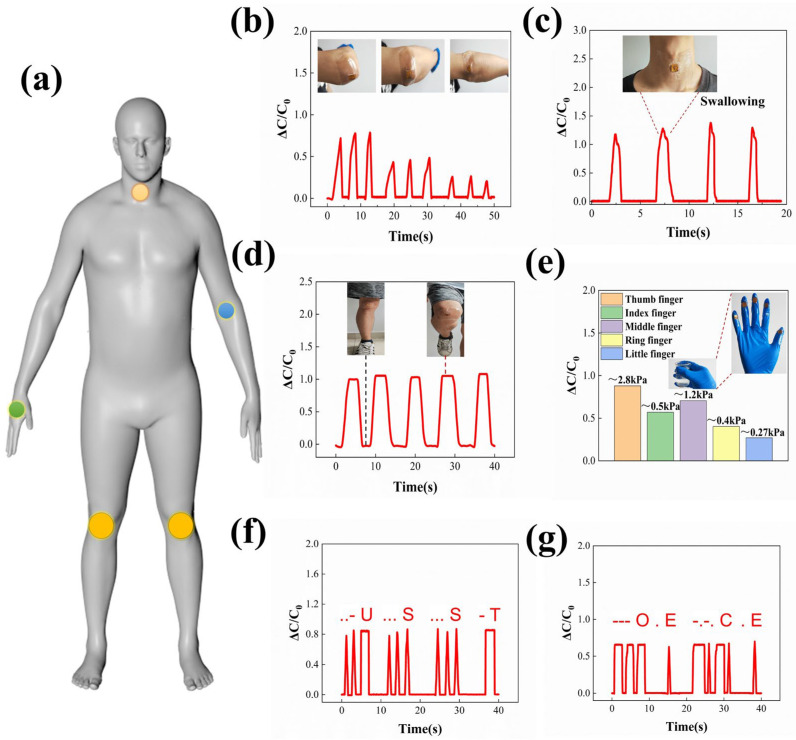
(**a**) Detection of human physiological activities at various locations on the body through the utilization of capacitive sensor pPDMS-h_2_. (**b**) Variations in capacitance levels during the flexion of the elbow. (**c**) Tracking changes in relative capacitance during the act of swallowing. (**d**) The response of the sensor from a volunteer engaging in leg lifts. (**e**) The pressure applied by individual fingers on a cup of water while being held is measured. (**f**,**g**) Generating specific Morse code signals through the act of pressing the sensor with the fingers.

This study showcased the potential applications of the sensor developed for monitoring human physiological activities. The fluorescent point in Figure 6a indicates the specific site where the sensor was deployed to monitor the volunteers’ physiological activities. By affixing the sensor to the elbow joint of the volunteers, it was feasible to track the degree of flexion in their arms. In Figure 6b, it is evident that the capacitive output is optimized when the arm assumes a 90° flexion, followed by a decrease in this response as the arm bends to a 45° angle. Even at minimal bending, a response is still generated. Swallowing, a critical physiological function, was examined by affixing the sensor to the volunteer’s throat during normal swallowing activity. Real-time capacitance data collected from the sensor revealed its responsiveness to the activity, including differences in swallowing speed and strength (Figure 6c). The sensor was affixed to the volunteer’s knee prior to engaging in leg-raising movements. As shown in Figure 6d, subsequent repetitive stretching and bending activities elicited periodic changes in the sensor’s response. To quantify the pressure applied by each finger on the water cup, sensors were affixed to the glove worn by the participant. The volunteer subsequently lifted the water cup, allowing for the measurement of pressure through the detection of capacitance value changes in the sensors on each finger. Analysis of the data, as depicted in Figure 6e, reveals that the thumb exerts the highest pressure on the water cup, while the little finger exerts the least pressure. Morse Code, a signaling code characterized by alternating signals, is utilized to convey various alphabets, numbers, and punctuation marks through unique sequences. This code serves a crucial function in radio communication, navigation, emergency signaling, and other domains. Volunteers attempted to transmit Morse code through the actuation of sensors, and the resulting Morse code signal was successfully detected on the testing equipment. The results of this experiment are depicted in Figure 6f,g, showcasing the successful transmission of the messages “USST” and “OECE” by the volunteers. The aforementioned applications were effectively demonstrated through the utilization of the sensor developed in this study, thereby showcasing the significant potential applications of this sensor in the realm of human body monitoring and signal transmission.

## 4. Conclusions

The objective of this study is to introduce a new flexible capacitive pressure sensor fabricated through the utilization of laser ablation and sacrificial templates. Initially, a PDMS film with approximately 46% porosity was produced using the sacrificial template technique. Subsequently, a 6 × 6 array of perforations was created on the porous PDMS film via a CO_2_ laser, leading to the development of the dielectric layer employed in this investigation. The preparation method described is cost-effective, environmentally sustainable, and easily manageable, allowing for the customization of array patterns and demonstrating significant potential for various applications. Through optimization of the ablation power of the CO_2_ laser and careful regulation of the number of ablations, we were able to identify the specific ablation parameters necessary to achieve the desired hole depth. Additionally, our research delved into the impact of varying spacing between adjacent holes within the array on sensor sensitivity, attributing differences in sensitivity to variations in Young’s modulus. Our research revealed a positive correlation between the proximity of neighboring cavities and sensor sensitivity, within the limitations of the machining process. The top-performing sensor in our study demonstrated a sensitivity of 0.694 kPa^−1^ within the range of 0–1 kPa. Additionally, the sensor exhibited a broad pressure detection range, remaining responsive even at pressures as high as 200 kPa and detecting pressures as low as 3 Pa. Furthermore, the device maintained consistent performance after undergoing 500 consecutive pressure loadings and unloadings. In order to showcase the practical utility of the sensor, we utilized it to effectively identify signals of human physiological activity and explored its capacity to convey pertinent information. These findings underscore the promising application prospects of the capacitive sensor developed by our team, thus holding significant implications for its broader implementation in the future.

## Figures and Tables

**Table 1:** A review of the performance specifications of several types of porous capacitive pressure sensors (NR = Not Reported).

Electrodes/Dielectric Layer	Key Materials to Fabricate the Dielectric	Pressure Range	Sensitivity	Response Time	Reference
AgNPs-SBS/ Microporous PDMS	PDMS/Glucose particles	0–2 kPa	0.278 kPa^−1^	340 ms	[63]
AgNWs and CFs-PDMS/ Microporous ecoflex	Ecoflex/Sugar	0–10 kPa	0.161 kPa^−1^	NR	[48]
ITO coated flexible PET/ Porous PDMS	PDMS/Sugar/Salt particles	0–5 kPa	0.171 kPa^−1^	162 ms	[66]
Ag-TPU/ microporous PDMS	PDMS/NaHCO_3_/ HNO_3_	0–50 Pa 0.2–1 MPa	0.3 kPa^−1^ 3.2 MPa^−1^	116 ms	[49]
ITO coated flexible PET/porous PDMS	PDMS/Deionized water	0.1–0.5 kPa	0.095 kPa^−1^	110 ms	[67]
CB-PDMS/ porous PDMS	PDMS/Citric acid monohydrate	0–4 kPa 4–14 kPa	0.1 kPa^−1^ 0.049 kPa^−1^	80 ms	[68]
PI-Cu/ microporous PDMS with hole array	PDMS/Salt microparticles	0–1 kPa 1–10 kPa 10–200 kPa	0.694 kPa^−1^ 0.077 kPa^−1^ 0.01 kPa^−1^	96 ms	This work

## Data Availability

All data from this work have been included in the article.

## Supplementary Materials

The following supporting information can be downloaded at: https://www.mdpi.com/article/10.3390/polym16162369/s1, Figure S1: Depth of ablation in porous PDMS dielectric layer as a function of CO_2_ laser power, at a scanning rate of 200 mm/s; Figure S2: (a) Physical illustration of a flexible capacitive sensor. (b–d) The exhibition of the mechanical flexibility of the sensor designed in this work, the upper right image representing the overall flexibility of the sensor. The two figures below show the flexibility of the electrodes and the dielectric layer, respectively; Figure S3: The circuit connection diagram of each experimental device during the performance test of the capacitive pressure sensor; Figure S4: Optical microscopy image of the cross-section of the dielectric layer of the sensors we designed during the hole depth optimization. (a) Optical microscopy image of the cross-section of the dielectric layer of the sensor pPDMS-h_1_. (b) Optical microscopy image of the cross-section of the dielectric layer of the sensor pPDMS-h_2_. (c) Optical microscopy image of the cross-section of the dielectric layer of the sensor PDMS-h_2_. (d) Hole depths obtained by laser ablation of bulk PDMS, performed three times at a laser power of 30 W and a scanning speed of 200 mm/s.; Figure S5: Relationship between pressure and compressive strain for capacitive sensors based on PDMS, pPDMS, pPDMS-h_1_, and pPDMS-h_2_ dielectric layers. Figure S6: The show of the actual drawings of three dielectric layers designed in the process of hole spacing optimization. (a–c) The three figures show, from left to right, the top views of the dielectric layer of the sensors pPDMS-w_0.8_, pPDMS-w_1.0_, and pPDMS-w_1.2_, respectively.
