# Supplementary material for: Highly Sensitive and Flexible Capacitive Pressure Sensors Combined with Porous Structure and Hole Array Using Sacrificial Templates and Laser Ablation
Source: Polymers (Basel). 2024 Aug 21;16(16):2369. doi: 10.3390/polym16162369 (PMC11359779; doi:10.3390/polym16162369)
Supplement: Supplementary file 1 [file polymers-16-02369-s001.zip › supplementary materials.pdf]

Supporting Information

Highly Sensitive and Flexible Capacitive Pressure Sensors Combined with Porous Structure and Holes Array by Sacrificial Templates and Laser Ablation

Yibin Zhao [1,2], Jingyu Zhou [1,2], Chenkai Jiang [1,2], Tianlong Xu [1,2], Kaixin Li [1,2], Dawei Zhang [1,2] and Bin Sheng [1,2,*]

1 School of Optical Electrical and Computer Engineering, University of Shanghai for Science and Technology, Shanghai 200093, China; 2135050829@st.usst.edu.cn (Y.Z.); 213330579@st.usst.edu.cn (J.Z.); 15003438539@163.com (C.J.); 223330649@st.usst.edu.cn (T.X.); 233350670@st.usst.edu.cn (K.L.); dwzhang@usst.edu.cn (D.Z.)

2 Shanghai Key Laboratory of Modern Optical Systems, Engineering Research Center of Optical Instruments and Systems, Shanghai 200093, China.

* Correspondence: bsheng@usst.edu.cn

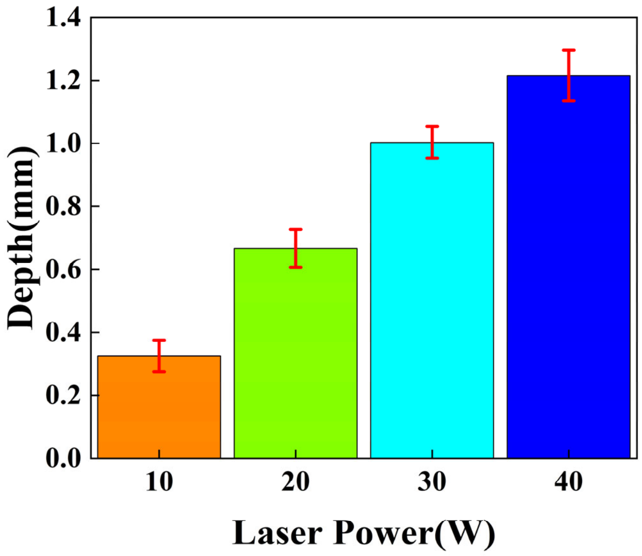

Figure S1. Depth of ablation in porous PDMS dielectric layer as a function of CO_2 laser power, at a scanning rate of 200 mm/s.

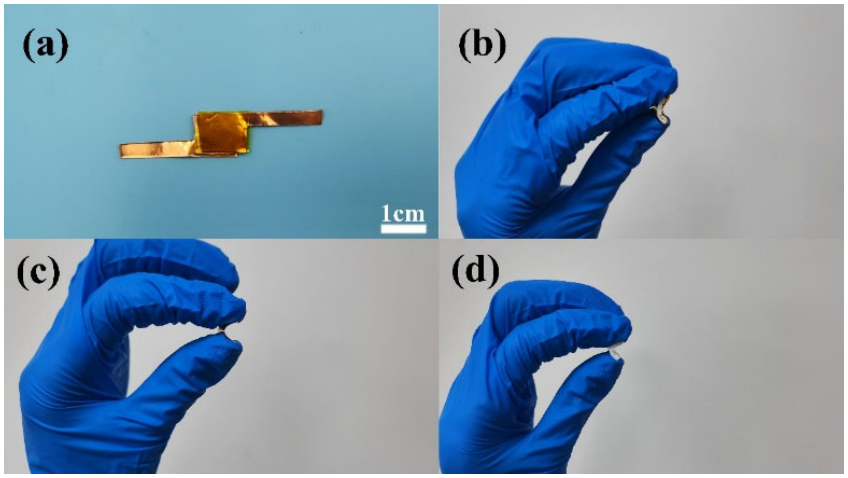

Figure S2. (a) Physical illustration of a flexible capacitive sensor. **(b-d)** The exhibition of the mechanical flexibility of the sensor designed in this work. The upper right image representing the overall flexibility of the sensor. The two figures below show the flexibility of the electrodes and the dielectric layer, respectively.

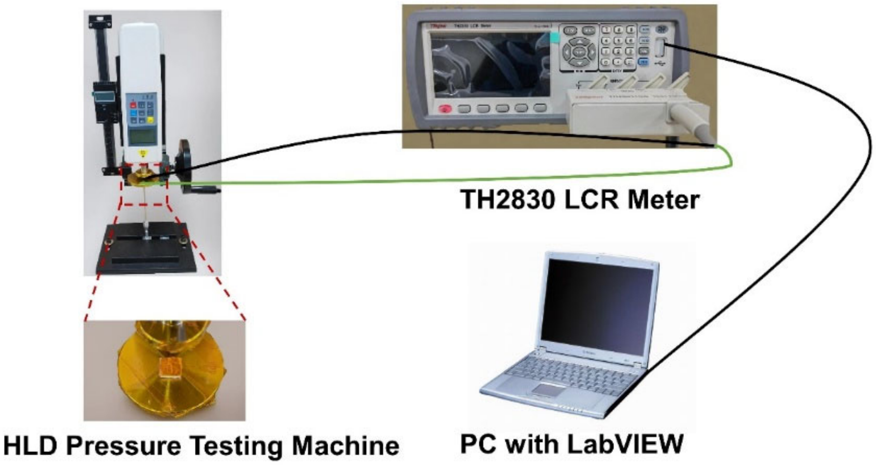

Figure S3. The circuit connection diagram of each experimental device during the performance test of capacitive pressure sensor.

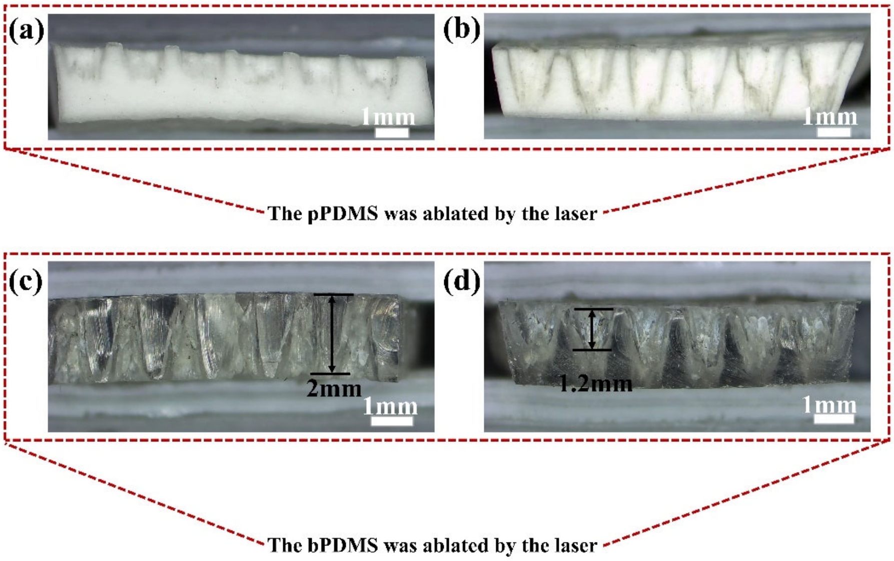

Figure S4. (a) Optical microscopy image of the cross-section of the dielectric layer of the sensor pPDMS-h_1. **(b)** Optical microscopy image of the cross-section of the dielectric layer of the sensor pPDMS-h_2. **(c)** Optical microscopy image of the cross-section of the dielectric layer of the sensor PDMS-h_2. **(d)** Hole depths obtained by laser ablation of bulk PDMS, performed three times at a laser power of 30 W and a scanning speed of 200 mm/s.

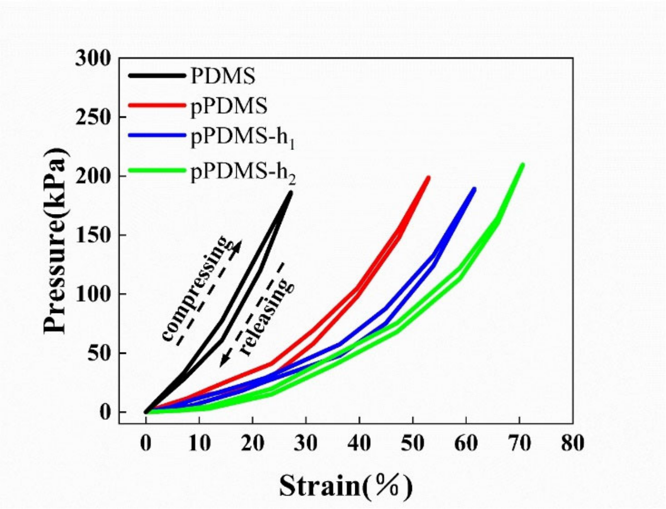

Figure S5. Relationship between pressure and compressive strain for capacitive sensors based on PDMS, pPDMS, pPDMS-h1, pPDMS-h2 dielectric layers.

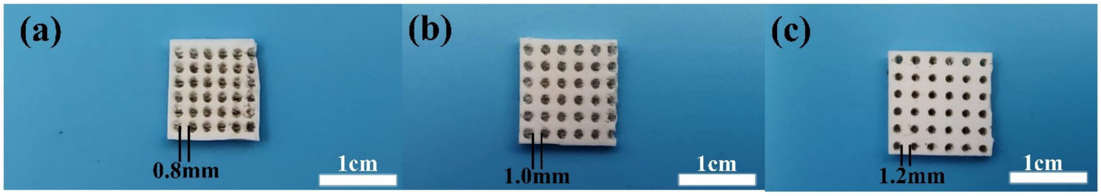

Figure S6. Three dielectric layers designed during the hole spacing optimization process. **(a-c)** The three figures show, from left to right, the top views of the dielectric layer of the sensors pPDMS-$w_{0.8}$, pPDMS-$w_{1.0}$, and pPDMS-$w_{1.2}$, respectively.
